# Endoscopic treatment of pancreaticopleural fistulas

**DOI:** 10.3389/fcimb.2022.939137

**Published:** 2022-10-03

**Authors:** Mateusz Jagielski, Jacek Piątkowski, Marek Jackowski

**Affiliations:** Department of General, Gastroenterological and Oncological Surgery, Collegium Medicum Nicolaus Copernicus University, Toruń, Poland

**Keywords:** pancreaticopleural fistula, pancreatic fistula, transpapillary drainage, transmural drainage, pancreatitis, endotherapy

## Abstract

**Introduction:**

Pancreaticopleural fistula (PPF) is a serious complication of acute and chronic pancreatitis.

**Objective:**

To evaluate the effectiveness of various endoscopic techniques for the treatment of patients with PPFs.

**Methodology:**

Prospective analysis of the results of endoscopic treatment of 22 patients with PPF due to pancreatitis was conducted at the Department of General, Gastroenterological, and Oncological Surgery, Ludwik Rydygier Collegium Medicum in Bydgoszcz, Nicolaus Copernicus University in Torun, between 2018 and 2021.

**Results:**

PPF was diagnosed in 22 patients (21 men and 1 woman; mean age 49.52 [30–67] years) with pancreatitis. In 19/22 (86.36%) patients, PPF communicated with the left pleural cavity and in 3/22 (13.64%) patients with the right pleural cavity. Chronic pancreatitis was diagnosed in 14/22 (63.64%) patients. Symptomatic pancreatic fluid collections were found in 15/22 (68.18%) patients with PPF (pancreatic pseudocyst in 11 and walled-off pancreatic necrosis in four patients). Endoscopic retrograde cholangiopancreatography was performed in 21/22 (95.45%) patients, confirming the diagnosis of PPF. All 21 patients underwent endoscopic sphincterotomy with prosthesis implantation in the main pancreatic duct (passive transpapillary drainage). In 1/22 (4.55%) patients, active transmural/transgastric drainage of the PPF was necessary due to inflammatory infiltration of the peripapillary region, precluding endoscopic pancreatography. Endoscopic transmural drainage was performed in all the 15 patients with pancreatic fluid collection. Clinical success was achieved in 21/22 (95.45%) patients. The mean total time of endotherapy was 191 (range 88–712) days. Long-term success of endoscopic treatment of PPFs during one year follow-up period was achieved in 19/22 (86.36%) patients.

**Conclusions:**

Endoscopic treatment is effective for managing post-inflammatory PPFs. The preferred treatment method is passive transpapillary drainage (prosthesis of the main pancreatic duct). If transpapillary drainage is not feasible, transmural drainage of the PPF remains the preferred method. Endoscopic transmural drainage leads to closure of the fistula canal in patients with pancreatic fluid collection complicated by PPF.

## Introduction

Fistulation, that is, the formation of pancreatic fistulas (PFs), may occur in the course of acute or chronic pancreatitis as the inflammatory process spreads (Jagielski et al., 2018a; Jagielski et al., 2018b; Larsen and Kozarek, 2014). A PF is an abnormal connection of the pancreatic ducts with another epithelium-covered surface, that is, with another organ, structure, or anatomical space (Larsen and Kozarek, 2014; Jagielski et al., 2018a; Jagielski et al., 2018a; Bassi et al., 2005; Morgan and Adams, 2007; Butturini et al., 2008). Regardless of the etiology, disruption of the main pancreatic duct (MPD) or smaller pancreatic ducts, defined as a break in the continuity of the duct leading to leakage of pancreatic juice, is at the root of the development of PF (Larsen and Kozarek, 2014; Jagielski et al., 2018a; Jagielski et al., 2018b). Disruption of MPD occurs in over 80% patients with post-inflammatory pancreatic and peripancreatic fluid collections (PPFCs) during the course of acute or chronic pancreatitis (Tay and Chang, 2013; Jagielski et al., 2017; Jagielski et al., 2020; Jagielski and Jackowski, 2021).

Pancreaticopleural fistula (PPF) is a rare complication of pancreatitis resulting from disruption of the MPD and leakage of pancreatic juice into the pleural cavity (Ali et al., 2009; Tay and Chang, 2013; Ramahi et al., 2019). Unlike pleural effusions seen in pancreatitis, which are usually clinically insignificant, PPFs often cause large, recurring pleural effusions (Ali et al., 2009).

Endoscopic treatment of disruption in the continuity of the MPD, and consequently, PFs caused by pancreatitis, involves performing endoscopic retrograde cholangiopancreatography (ERCP) with endoscopic sphincterotomy and implantation of the prosthesis into the MPD (passive transpapillary drainage) to ensure physiological outflow of pancreatic juice into the duodenum (Jagielski et al., 2017; Jagielski et al., 2018a; Jagielski et al., 2018b; Jagielski et al., 2020; Jagielski and Jackowski, 2021b)

The use of endoscopic techniques in the treatment of MPD disruption caused by pancreatitis remains controversial (Boxhoorn et al., 2021). Most of the evidence on the diagnosis and therapy of post-inflammatory PPFs is derived from single case reports (Wee et al., 2017; Ramahi et al., 2019). Moreover, the management of patients with pancreatitis and hindered access to the MPD through the major duodenal papilla during ERCP due to swelling of the duodenal wall or altered anatomy of the upper gastrointestinal tract remains challenging.

Therefore, this study presents the results of treatment of patients with PPF due to pancreatitis using various endoscopic techniques.

## Materials and methods

This was a prospective analysis of treatment outcomes in patients with pancreatitis hospitalized at the Department of General, Gastroenterological, and Oncological Surgery, Ludwik Rydygier Collegium Medicum in Bydgoszcz, Nicolaus Copernicus University in Torun between 2018 and 2021. A significant number of these patients had previously been treated for pancreatitis in other clinical centers and were subsequently transferred to our referral center to treat the sequelae and complications of pancreatitis (Jagielski and Jackowski, 2021b).

The study was approved by the Ethics Committee at the Collegium Medicum of Nicolaus Copernicus University and was conducted in accordance with the Declaration of Helsinki. All patients provided oral and written informed consent to participate in the study. All patients received detailed information regarding the study.

The diagnosis of pancreatitis, the criteria of clinical and morphological categorization, and all definitions of local and systemic complications were based on the 2012 revised Atlanta classification (Sarr et al., 2013; Thoeni, 2012; Banks et al., 2013). The standards for conservative treatment of pancreatitis were based on international guidelines (Tenner et al., 2013; Working Group IAP/APA Acute Pancreatitis Guidelines, 2013). Conservative treatment relies primarily on dietary treatment with intensive intravenous fluid therapy and analgesia. Moreover, additional treatment methods were used depending on concomitant organ impairment and the patient’s overall clinical condition. The decision to use interventional treatment for complications of pancreatitis was made after careful consideration of the clinical picture and imaging results, mostly contrast-enhanced abdominal computed tomography (CECT) images. In the case of qualification for interventional treatment, endoscopic techniques are the method of choice at our center (Jagielski and Jackowski, 2021a; Jagielski and Jackowski, 2021b).

### Study inclusion criteria

All symptomatic patients with PPFs in the course of acute or chronic pancreatitis were included in the study. Qualification for endoscopic treatment was based on the clinical picture and imaging results, mainly based on CECT of the abdomen and magnetic resonance imaging (MRI).

### Study exclusion criteria

Patients with PPFs without clinical signs associated with the presence of a fistula or with PPFs that were not a consequence of pancreatic inflammatory disease (acute or chronic pancreatitis) were excluded from the study. Patients who underwent surgery in the pancreatic region were also excluded.

### Management strategy in patients with post-inflammatory pancreaticopleural fistula

Pleural fluid puncture with determination of amylase levels and passive drainage of the pleural cavity was performed in all patients with pancreatitis and suspected PPF based on the clinical picture and imaging. If pleural amylase activity exceeded 1000 U/l, the patient was diagnosed with PPF and referred for endoscopic treatment with transpapillary ERCP (through the major duodenal papilla). If transpapillary access was not possible, transmural access (through the wall of the upper gastrointestinal tract) was obtained under endoscopic ultrasound (EUS) guidance. Endoscopic drainage of the collection was performed in patients with PPFs and PPFCs. Somatostatin infusion was administered at a dose of 3.5 micrograms/kg body weight/hour from the time of diagnosis of the PPF until completion of pleural drainage.

### Endoscopic procedures

Endoscopic procedures were performed under general anesthesia with tracheal intubation. All patients provided informed consent for the endoscopic procedures. All procedures were performed by a single endoscopist, and entailed carbon dioxide insufflation and the use of a linear echoendoscope (Pentax EG3870UTK, Pentax Medical, Tokyo, Japan), duodenoscope (Olympus TJF-Q180V, Olympus Corporation, Tokyo, Japan), and gastroscope (Olympus GIF-H185, Olympus Corporation) [16].

#### Transpapillary drainage

**Figure 1:**
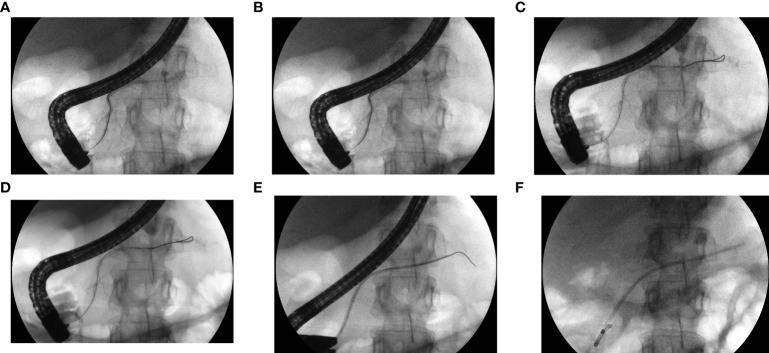
**(A–F)**. ERCP with passive transpapillary drainage. The patient with MPD partial disruption (PPF) in pancreatic tail. Contrast medium and guidewire were introduced to MPD during ERCP **(A–C)**. MPD partial disruption in form of PPF in pancreatic tail became visible as leakage of contrast beyond the MPD **(A–C)**. During subsequent steps of ERCP the pancreatic stent was introduced along the guidewire **(D, E)**. Pancreatic stent created bridged the partial disruption of MPD in pancreatic tail **(F)**.

Attempts to perform ERCP to assess the morphology and integrity of the MPD and to employ possible endoscopic treatment were made in all patients with post-inflammatory PPF treated in our center (**Figures 1A–F**). In the case of disruption of the MPD, sphincterotomy (Fusion OMNI Sphincterotome FS-OMNI-35-480, Cook Endoscopy Inc., North Carolina, USA) was performed and a pancreatic 5 Fr, 7 Fr, 8.5 Fr, or 10 Fr endoprosthesis (Zimmon Pancreatic Stent, Cook, Endoscopy Inc., North Carolina, USA) was introduced into the MPD and subsequently replaced every 1, 3, 6, 12, or 24 months or until no contrast leakage outside the duct was identified.

#### Transmural drainage

**Figure 2:**
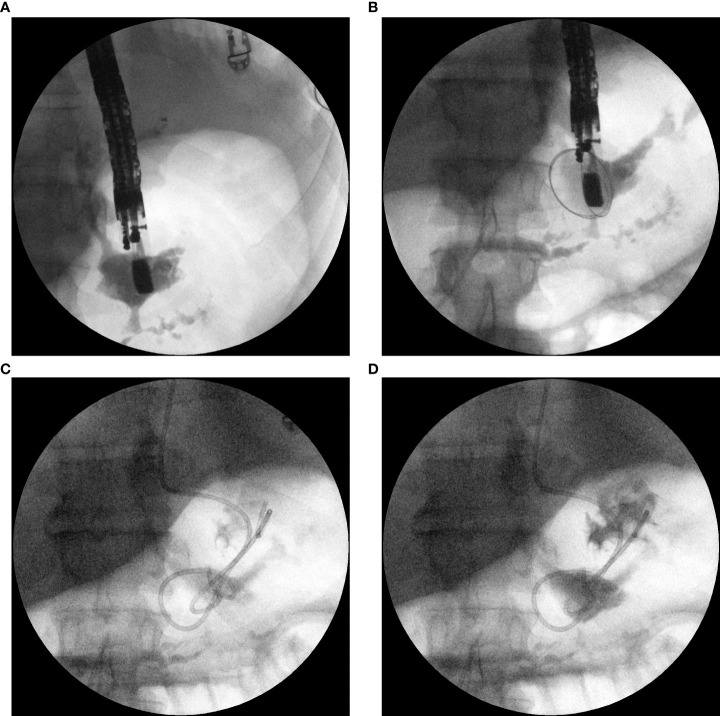
**(A–D)**. Transmural drainage of PPF. Antegrade endoscopic pancreatography. Fluoroscopic images taken during the endoscopic procedure after the transmural puncture of the PPF canal **(A, B)**. The administered contrast filled the pleural fistula with a visible infiltration of the contrast into the pancreatic duct by partial disruption to the MPD **(A, B)**. Transmural drainage of PPF- plastic transmural stent and nasal drain introduced through the transmural fistula is visible **(C, D)**. Contrast medium administered through the nasal drain filled the PPF canal and was leaking through the stent into stomach **(D)**.

If transpapillary access was not possible, transmural access (through the wall of the upper gastrointestinal tract) was obtained using the single transluminal gateway technique (SGT) (**Figures 2A–D**). Placement of the pancreaticogastric anastomosis in the form of a transmural cystostomy was performed under EUS guidance. Anastomosis between the lumen of the gastrointestinal tract and the fistula canal was created using a 10 Fr cystotome (Cystotome CST-10, Cook Endoscopy Inc., North Carolina, USA) and then dilated using a high-pressure balloon with a diameter of up to 15 mm (Cook Endoscopy Inc., North Carolina, USA). A transmural 7 Fr or 8 Fr double-pigtail stent (Cook Endoscopy Inc., North Carolina, USA) was inserted through pancreaticogastrostomy. For active transmural drainage, a 7 Fr or 8.5 Fr nasal drain (Cook Endoscopy Inc., North Carolina, USA) was inserted into the canal of the fistula through pancreaticogastric anastomosis. In cases of passive transmural drainage, only 7 Fr or 8 Fr double-pigtail stents (Cook Endoscopy Inc., North Carolina, USA) were used through the transmural anastomosis.

#### Postoperative period

In the postoperative period, all patients with post-inflammatory PPF continued to receive somatostatin as a continuous intravenous infusion at a dose of 3.5 micrograms/kg body weight/hour until pleural drainage was complete, i.e., when the amount of drained pleural fluid did not exceed 50 ml per day without underwater seal drain.

In patients with active transmural drainage of the PPF, the nasal drain was rinsed with 50 ml of saline every 4 h. Active transmural drainage was considered as completed at the end of pleural drainage, the nasal drain was removed, and a double-pigtail plastic transmural endoprosthesis was left for passive transmural drainage of the PPF.

CECT of the chest and abdominal cavity was performed at the time of completion of the endoscopic treatment. During follow-up after the end of endotherapy, control imaging tests (mainly CECT) were performed every 3, 6, 12, and 24 months and subsequently every two years if no symptoms were reported. During follow-up no patient developed contrast induced nephropathy.

### Definitions

Partial disruption of the MPD was defined as the flow of contrast, during ERCP, outside the MPD with contrast filling of the part of the duct distal to the disruption site.

Complete disruption of the MPD was defined as the flow of contrast outside the duct without contrast filling of the distal part of the MPD during ERCP.

Closure of the PPF was defined as a lack of visualization on imaging of a communication passage that existed earlier between the lumen of the pleural cavity and the MPD.

Complications of endoscopic treatment were divided into early complications (occurring up to 30 days after treatment), evaluated in line with the Clavien-Dindo classification (Clavien et al., 2009).

Clinical success of endoscopic treatment of post-inflammatory PPFs was defined as closure of the PPF, absence of pleural effusion, and lack of clinical signs associated with PPF.

The long-term success of endoscopic treatment of PPFs was defined as closure of the PPF, absence of pleural effusion, and lack of clinical signs associated with the PPF during the follow-up period.

### Statistics

All statistical calculations were performed using STATISTICA data analysis software (StatSoft Inc., 2014). Quantitative variables are presented as arithmetic means and minimal and maximal values (ranges), whereas qualitative data are presented as means of numbers and percentages.

## Results

A total of 882 (556 men, 326 women; mean age 54.44 [19–101] years) patients with pancreatitis were treated at the Department of General, Gastroenterological and Oncological Surg+ery, Ludwik Rydygier Collegium Medicum in Bydgoszcz, Nicolaus Copernicus University in Torun between 2018 and 2021.

Post-inflammatory PPF was diagnosed in 22/882 (2.49%) patients (21 men, 1 woman; average age 49.52 [30–67] years). None of the patients underwent any surgical intervention before a diagnosis of PPF was made. Chronic pancreatitis was diagnosed in 14/22 (63.64%) patients with PPF. The etiology of pancreatitis in this group of patients was alcohol-related in 16 patients and non-alcoholic in 6 (5 – biliary, 1 – iatrogenic). The average time from the beginning of the pancreatitis episode until the diagnosis of PPF was 52 (23–119) days. No further PFs were found in any of the patients. Detailed clinical characteristics of the patients are presented in **Table 1**.

**Table 1:** Characteristics of the patients from study group.

	All patients (n=22)
**Age**, mean [range] years	49.52 [30–67]
**Sex**, n men (%)	21 (95.45%)
**Chronic pancreatitis**, n (%)	14 (63.64%)
**Etiology of pancreatitis**, n (%)	
Alcoholic	16 (72.73%)
Non-alcoholic	6 (27.27%)
**Symptomatic PPFCs**, n (%)	15 (68.18%)
Pancreatic pseudocyst	11
Walled-off pancreatic necrosis	4
**CTSI** (computed tomography severity index), mean [range] points	6 [4-10]
**Time from the beginning of the pancreatitis episode until the diagnosis of PPF**, mean [range] days	52 [23–119]
**PPF localization,** n	
Pancreatic head	3
Pancreatic body	15
Pancreatic tail	4
**Symptoms related with PPF,** n	
Shortness of breath	17
Chest pain	11
Abdominal pain	13
Fever	4
**Method of minimally invasive treatment of PPF,** n (%)	
Transpapillary drainage (ERCP)	21 (95.45%)
Transmural drainage	1 (4.55)
**Complications of interventional treatment,** n (%)	
Upper gastrointestinal bleeding	3 (13.63%)
Sepsis	1 (4.55%)

Symptoms suggestive of PPFs were reported in 20/22 (90.91%) patients. Shortness of breath was reported by 17 patients, chest pain by 11 patients and abdominal pain by 13 patients. Furthermore, fever was observed in 4 patients in this group. Sepsis was diagnosed in 3 patients with PPF (*Escherichia coli* and *Enterococcus faecalis* were the most commonly grown pathogens). These symptoms are non-specific for PPFs.

Symptomatic PPFCs were found in 15/22 (68.18%) patients with PPF (pancreatic pseudocyst in 11 patients; walled-off pancreatic necrosis in 4 patients). The mean size of collection was 7.96 (5.44–16.3) cm. Infected PPFCs was revealed in 4/15 (26.67%) patients. The collection was initially sterile in 11/15 (73.34%) patients.

In all 22 patients, the diagnosis of PPF due to pancreatitis was made based on CECT of the chest, abdomen, and pelvis, as well as amylase high activity in the pleural fluid. The average amylase activity in the pleural fluid was 9883 (1221–230000) U/l. In 19/22 (86.36%) patients, PPF communicated with the left pleural cavity; in 3/22 (13.64%) patients with the right pleural cavity. Pleural cavity drainage was performed in all 22 patients. The mean drainage time was 5 (3–17) days.

All 22 patients with post-inflammatory PPFs were referred for endoscopic treatment. Transpapillary access to the PPF (anatomically through the major duodenal papilla) was achieved in 21 patients. In one patient, extraanatomical transmural/transgastric access was performed due to inflammatory infiltration of the peripapillary region of the descending duodenum, which prevented transpapillary access.

ERCP was performed in 21/22 (95.45%) patients, confirming the presence of PPF. Partial disruption of the MPD (pancreatic head, 3 patients; pancreatic body, 12 patients; pancreatic tail, 4 patients) communicating with the pleural cavity through a fistula was found in 19/21 (90.48%) patients. In the remaining 2/21 (9.52%) patients, complete disruption of the MPD was found in the pancreatic body. All 21 patients underwent endoscopic sphincterotomy with prosthesis implantation into the MPD (passive transpapillary drainage). The mean number of transpapillary replacements of the pancreatic endoprostheses was 2.55 (1–7). The mean total time endoscopic prostheses remained in the MPD was 191 (88–712) days.

In 1/22 (4.55%) patients, active transmural/transgastric drainage of the PPF was used because of inflammatory infiltration of the peripapillary region preventing ERCP. Active drainage of the fistula lasted for seven days, followed by passive drainage for the following 102 days.

Endoscopic transmural drainage, lasting 13 (4–36) days on average, was performed in all 15 patients with PPFC, followed by 72 (33–367) days of passive transmural drainage.

Procedure-related complications occurred in 4/22 (18.18%) patients. Three patients required transfusion of packed red blood cells because of gastrointestinal bleeding (Clavien–Dindo grade II). Sepsis requiring intravenous broad-spectrum antibiotic therapy (Clavien–Dindo grade II) was observed during endotherapy in one patient only. None of the patients required surgical treatment for complications of endotherapy.

Closure of PPF was confirmed by imaging studies and ERCP in 21/22 (95.45%) patients. The mean time from diagnosis to PPF closure was 66 (33–171) days.

Clinical success of endoscopic treatment of post-inflammatory PPFs was achieved in 21/22 (95.45%) patients. One patient out of the 22 (4.55%) patients was still undergoing endotherapy due to complete disruption of the MPD.

Recurrence of PPF was identified in two patients. One patient with recurrence of PPFC complicated by PPF required repeated endoscopic treatment. One patient required thoracosurgical treatment because of recurrence of a PPF complicated by pleural empyema. Long-term success of endoscopic treatment of PPFs during one year of follow-up was achieved in 19/22 (86.36%) patients.

## Discussion

The current literature lacks clear guidelines defining an algorithm for performing diagnostic and therapeutic procedures in patients with PPFs. Most of the data are derived from individual case reports or case series, described in this publication (Ali et al., 2009; Wee et al., 2017; Ramahi et al., 2019). The present study is the largest case series demonstrating the effectiveness of various endoscopic techniques in patients with post-inflammatory PPF available in the literature to date.

Post-inflammatory PPF is an uncommon but serious complication of acute and more often chronic alcohol-induced pancreatitis (Dhebri and Ferran, 2005; Ali et al., 2009; Tay and Chang, 2013; Wee et al., 2017; Ramahi et al., 2019). The precise incidence rate of PPF is unknown and its estimation remains difficult. According to the literature, PPF is diagnosed in approximately 0.4% of patients with pancreatitis (Dhebri and Ferran, 2005; Tay and Chang, 2013). In our study, the incidence rate of PPF in patients with pancreatitis was 2.49% (Dhebri and Ferran, 2005; Tay and Chang, 2013). However, this is difficult to interpret in the context of the general incidence rate of PPF in the entire population of patients with pancreatitis because our facility is a reference center for the minimally invasive treatment of inflammatory diseases of the pancreas. A significant number of patients from the study group had previously been treated in other clinical centers for pancreatitis and were transferred to our referral center for the treatment of the sequelae and complications of pancreatitis.

There are no typical clinical features of post-inflammatory PPF, making diagnosis difficult. Patients with post-inflammatory PPF often report severe abdominal pain as the predominant symptom, accompanied by shortness of breath and chest pain, which does not unequivocally point to the suspicion of post-inflammatory PPF based on the clinical picture alone. As shown in this study, abdominal pain typical of pancreatitis often masks chest symptoms, which are more characteristic of PPF. According to the available literature, shortness of breath is the most common clinical symptom associated with PPF, followed by abdominal pain, chest pain, cough, hemoptysis, weight loss, fever, and other non-specific symptoms (Ondrejka et al., 2000; Dhebri and Ferran, 2005; Oh et al., 2006; Ali et al., 2009; Tay and Chang, 2013; Wee et al., 2017; Ramahi et al., 2019).

Diagnostic difficulties in this group of patients represent one of the main problems in determining the exact incidence of PPF often delaying the correct diagnosis. Non-invasive magnetic resonance cholangiopancreatography (MRCP) is the most sensitive and specific diagnostic test for PFs resulting from disruption of the MPD. On the other hand, suspicion of PF due to MPD disruption is an indication for ERCP (Devière et al., 1995; Varadarajulu et al., 2005). In cases where MPD disruption and the presence of PPF are confirmed during ERCP, an endoprosthesis may be inserted into the MPD to secure physiological outflow of pancreatic juice into the lumen of the duodenum and subsequent PPF closure (Devière et al., 1995; Varadarajulu et al., 2005; Jagielski et al., 2018a; Jagielski et al., 2018b). It is also recommended that secretin-stimulated MRCP (secretin MRCP) should be performed to evaluate MPD when there is no suspicion of PPF as a result of MPD disruption and there is no need to apply endoscopic treatment (Matos et al., 1997; Soto et al., 2001; Punwani et al., 2003). Secretin MRCP is considered a safe and non-invasive imaging technique that enables visualization of the entire anatomy of the pancreas, including the pancreatic ducts (Matos et al., 1997; Soto et al., 2001; Punwani et al., 2003). Despite the availability of MRCP in our center, if PPF was suspected based on high amylase activity in the pleural fluid, we performed ERCP without confirming the diagnosis using MRCP. If the presence of PPF was confirmed during ERCP, endoscopic sphincterotomy was performed with implantation of a prosthesis into the MPD. This study demonstrates that such a diagnostic and therapeutic algorithm in patients with post-inflammatory PPF is associated with effective treatment and low likelihood of complications.

Disruption to the MPD or smaller pancreatic ducts, leading to leakage of pancreatic juice outside the pancreatic ducts, is the most important factor in the pathophysiology of PFs (Larsen and Kozarek, 2014; Jagielski et al., 2018a; Jagielski et al., 2018b). Disruption of the pancreatic ducts on the anterior surface of the pancreas usually leads to the development of a PPF and manifests as pancreatic ascites. Disruption of the pancreatic ducts on the posterior surface of the pancreas leads to the formation of a PPF with pancreatic juice leaking into the pleural cavity. In the case of PFs, pancreatic juice usually spreads retroperitoneally to pleural cavities through the paths of least resistance, that is, through the aortic or esophageal hiatus (Dhebri and Ferran, 2005; Ali et al., 2009; Tay and Chang, 2013). Transdiaphragmatic communication is very rare (Dhebri and Ferran, 2005; Ali et al., 2009; Tay and Chang, 2013).In most patients with PPFs, fluid is found in the left pleural cavity, and less often on the right side or bilaterally (Ondrejka et al., 2000; Dhebri and Ferran, 2005; Oh et al., 2006; Ali et al., 2009; Tay and Chang, 2013).As presented above, with post-inflammatory PPFs, pancreatic juice can leak directly into the pleural cavities from the site of the pancreatic ducts disruption. The pathophysiology is different in patients with post-inflammatory PPFC complicated by PPF. In such cases, pancreatic juice leaks into the lumen of the collection, which communicates with the pleural cavity through a PF. In this study, symptomatic PPFCs were found in most patients with PPFs caused by pancreatitis, the most common finding being a pancreatic pseudocyst in the course of chronic pancreatitis.

In this study, it was shown that in patients with PPFCs complicated by PPF, endoscopic transmural drainage of the collection resulted in closure of the fistula canal. Similar observations were found in a group of patients with PPFCs complicated by pancreaticocolonic fistula, in which effective drainage of the collection resulted in regression of the intestinal fistula (Jagielski et al., 2018).

In the largest study on the endotherapy of post-inflammatory PPFs available in the literature, Wroński et al. presented the results of treatment of eight patients (Wronski et al., 2011). Endoscopic treatment was applied during ERCP in seven patients; in one patient, the major duodenal papilla could not be found and cannulation failed, and the patient underwent surgical treatment (Wronski et al., 2011). In our study, 22 patients with post-inflammatory PPFs were treated endoscopically. Transpapillary drainage was performed during ERCP in 21 patients; in one patient, the duodenal papilla major could not be accessed, and effective extraanatomical (transmural) drainage of the PPF was performed. Thus, it was demonstrated that surgical treatment can be prevented through the application of advanced endoscopic techniques such as extraanatomical transgastric drainage.

In the aforementioned work, Wroński et al. (Wronski et al., 2011) noted the technical success of the ERCP procedure in seven (87.5%) patients, although three patients required subsequent surgery due to failed endoscopic treatment (ineffective transpapillary drainage and superinfection of the pleura or pancreatic collections). Ultimately, clinical success of endotherapy was achieved in 4 (50%) patients (Wronski et al., 2011). In our study, technical success of endoscopic surgery was achieved in all 22 patients. However, clinical success of endotherapy was achieved in 21 (95.45%) patients, and long-term success of endoscopic treatment of PPFs was noted in 19 (86.36%) patients.

In our opinion, the poor results of endoscopic treatment reported by Wroński et al. (Wronski et al., 2011) are associated with difficult anatomical conditions due to chronic pancreatitis found during ERCP in the study population. In only one (12.5%) patient, it was possible to properly introduce pancreatic endoprosthesis during ERCP so that it covered the site of disruption (Wronski et al., 2011). In the remaining six patients, placement of the pancreatic stent failed because intraductal stones and ductal strictures precluded its passage or the stent was too short to reach the fistula located in the distal part of the pancreas (Wronski et al., 2011). In our study, no such abnormalities were encountered during ERCP, which would prevent the proper introduction of a pancreatic endoprosthesis. Moreover, Wroński et al. (Wronski et al., 2011) showed that in patients with post-inflammatory PPF, complete disruption of the MPD in the pancreatic body and leakage into the left pleural cavity were the most common findings. In our study, both the body and tail were the most common locations of PPF with leakage into the left pleural cavity. However the majority (90.48%) of patients suffered from partial disruption of the MPD, which enabled stenting of the disruption using pancreatic endoprosthesis and, consequently, led to better results of endoscopic treatment. Partial disruption of the MPD is associated with better outcomes of endotherapy compared to complete disruption of the MPD (Devière et al., 1995; Varadarajulu et al., 2005; Jagielski et al., 2017; Jagielski et al., 2018a; Jagielski and Jackowski, 2021a). Although endotherapy is a more effective treatment method in cases of partial disruption of the pancreatic duct compared with total disruption in patients with pancreatitis, we believe that stenting of the MPD should also be applied in patients with complete duct disruption (Jagielski et al., 2018b; Jagielski et al., 2021a; Jagielski et al., 2017). However, it should be noted that most patients with complete MPD disruption require permanent passive transmural drainage in addition to passive transpapillary drainage (stenting of the MPD), especially in cases of pancreatic fragmentation (disconnected duct syndrome) (Jagielski et al., 2017; Jagielski et al., 2018a; Jagielski et al., 2018b; Jagielski and Jackowski, 2021a).

The results of our study make an important contribution to the current state of knowledge, as there is currently no consensus regarding the optimal treatment for patients with post-inflammatory PPFs. According to literature, conservative treatment should be the first-line treatment (Dhebri and Ferran, 2005; Ali et al., 2009; Tay and Chang, 2013). Interventional treatment using minimally invasive techniques may only be initiated if conservative management is ineffective (Dhebri and Ferran, 2005; Ali et al., 2009; Tay and Chang, 2013). In the case of ineffective minimally invasive methods, surgery remains the treatment of choice (Dhebri and Ferran, 2005; Ali et al., 2009; Tay and Chang, 2013). The diagnostic and therapeutic algorithms presented in this study appear optimal for patients with post-inflammatory PPFs. In patients with confirmed pancreatitis, the detection of a pleural effusion on imaging requiring thoracocentesis with high amylase activity in the drained fluid is sufficient for the diagnosis of post-inflammatory PPF and for the implementation of treatment, particularly when this coexists with post-inflammatory PPFCs. In our study, the first-line treatment in patients with post-inflammatory PPF was endotherapy in combination with pharmacotherapy. In the literature, treatment often begins with conservative management, and a decision to perform ERCP is made only where conservative management is ineffective (Tay and Chang, 2013; Dhebri and Ferran, 2005; Ali et al, 2009). In contrast to previous reports, in this study, ERCP was performed in all patients with post-inflammatory PPFs, which confirms the diagnosis and also allows the implementation of treatment to decompress the pancreatic duct system and restore the outflow of pancreatic juice to the duodenum.

The main limitations of our study include the lack of randomization and the fact that the study was conducted on a selected group of patients from a single center. Although our study presents the experience of a single center, the fact that all endoscopic procedures were performed by a single endoscopist may be considered a strength of the study as this enables a reliable comparison of the results of endoscopic treatment.

In summary, regardless of the clinical situation, the management of patients with post-inflammatory PPFs should begin with the use of minimally invasive techniques, often combined with intensive conservative treatment. The study showed that endoscopic techniques, such as minimally invasive treatment, in patients with post-inflammatory PPF are effective. Transpapillary drainage (stenting of the MPD) was the preferred method. If transpapillary drainage is not possible, transmural drainage of the PPF remains the management of choice. In patients with PPFS complicated by PPF, drainage of the collection through endoscopic transmural drainage leads to closure of the fistula canal.

## Data availability statement

The raw data supporting the conclusions of this article will be made available by the authors, without undue reservation.

## Ethics statement

The study was approved by the Ethics Committee at the Collegium Medicum of Nicolaus Copernicus University and was conducted in accordance with the Declaration of Helsinki. All patients provided oral and written informed consent to participate in the study. All patients received detailed information regarding the study.

## Author contributions

Conceptualization was performed by MatJ, JP and MarJ. Formal analysis was performed by MatJ, JP, and MarJac. The methodology was performed by MatJ, JP and MarJ. Project administration was performed by MatJ. Endoscopic procedures was performed by MatJ. The writing of the original draft was performed by MatJ, and JP. Editing was performed by MatJ, and MarJ. All authors (MatJ, JP, and MarJ) revised and approved the submitted version of the manuscript.

## Conflict of interest

The authors declare that the research was conducted in the absence of any commercial or financial relationships that could be construed as a potential conflict of interest.

## Publisher’s note

All claims expressed in this article are solely those of the authors and do not necessarily represent those of their affiliated organizations, or those of the publisher, the editors and the reviewers. Any product that may be evaluated in this article, or claim that may be made by its manufacturer, is not guaranteed or endorsed by the publisher.
